# Update to the FOCUS, AFFINITY and EFFECTS trials studying the effect(s) of fluoxetine in patients with a recent stroke: statistical analysis plan for the trials and for the individual patient data meta-analysis

**DOI:** 10.1186/s13063-020-04875-1

**Published:** 2020-11-25

**Authors:** Gillian Elizabeth Mead, Catriona Graham, Laurent Billot, Per Näsman, Erik Lundström, Steff Lewis, Graeme J. Hankey, Maree L. Hackett, John Forbes, Martin Dennis, On behalf of the FOCUS, AFFINITY and EFFECTS trialists

**Affiliations:** 1 Usher Institute, University of Edinburgh, , Room S1642, Royal Infirmary, Little France Crescent, Edinburgh, EH16 4SA UK; 2 Welcome Trust Clinical Research Facility, Western General Hospital, Crewe Road, Edinburgh, EH4 2XU UK; 3 The George Institute for Global Health, University of Sydney, Sydney, NSW Australia; 4 Center for Safety Research, KTH Royal Institute of Technology, Stockholm, Sweden; 5 Department of Neuroscience, Neurology, Uppsala University, Uppsala, Sweden; 6 University of Edinburgh, Level 2, NINE Edinburgh BioQuarter, 9 Little France Road, Edinburgh, EH16 4UX UK; 7 Medical School, Faculty of Health and Medical Sciences, The University of Western Australia, Crawley, WA Australia; 8 Health Research Institute, University of Limerick, Limerick, Ireland; 9 Centre for Clinical Brain Sciences, University of Edinburgh, Chancellor’s Building, Little France Crescent, Edinburgh, EH16 4SA UK

**Keywords:** Stroke, Recovery, Individual patient data meta-analysis, FOCUS, AFFINITY, EFFECTS

## Abstract

**Background:**

Three large trials of fluoxetine for stroke recovery (FOCUS (fluoxetine or control under supervision), AFFINITY (the Assessment oF FluoxetINe In sTroke recovery) and EFFECTS (Efficacy oF Fluoxetine—a randomisEd Controlled Trial in Stroke)) have been collaboratively designed with the same basic protocol to facilitate an individual patient data analysis (IPDM). The statistical analysis plan for the three individual trials has already been reported in *Trials*, including a brief description of the IPDM. In this protocol, we describe in detail how we will perform the IPDM.

**Methods/design:**

Data from EFFECTS and AFFINITY will be transferred securely to the FOCUS statistician, who will perform a one-stage IPDM and a two-stage IPDM. For the one-stage IPDM, data will be combined into a single data set and the same analyses performed as described for the individual trials. For the two-stage IPDM, the results for the three individual trials will be combined using fixed effects meta-analyses.

The primary and secondary outcome domains for the IPDM are the same as for individual trials. We will also perform analyses according to several subgroups including country of recruitment, ethnicity and trial. We will also explore the effects of fluoxetine on our primary and secondary outcomes in subgroups defined by combinations of characteristics.

We also describe additional research questions that will be addressed using the combined data set, and published subsequently, including predictors of important post-stroke problems such as seizures, low mood and bone fractures.

**Discussion:**

An IPDM of our three large trials of fluoxetine for stroke recovery will allow us to provide the most precise estimates of any risks and benefits of fluoxetine vs placebo, to detect reliably a smaller overall effect size than those detectable by the individual trials, to better determine the effects of fluoxetine vs placebo in subgroups of patients and outcomes and to broaden the generalisability of the results. Also, we may identify differences in treatment effects between studies.

**Trial registration:**

FOCUS: ISRCTN ISRCTN83290762. Registered on 23 May 2012. EudraCT 2011-005616-29. Registered on 3 February 2012.

AFFINITY: Australian New Zealand Clinical Trials Registry ACTRN12611000774921. Registered on 22 July 2011.

EFFECTS: ISRCTN ISRCTN13020412. Registered on 19 December 2014. ClinicalTrials.gov NCT02683213. Registered on 2 February 2016. EudraCT 2011-006130-16. Registered on 8 August 2014.

## Background

### Burden of stroke

Despite recent advances in hyperacute treatments and secondary prevention, stroke is still a major cause of death and severe disability in the community [1, 2]. In low- and middle-income countries, the burden of stroke is increasing. Thus, further research is needed to identify new treatments that will reduce the burden of stroke disease globally.

### Fluoxetine and stroke recovery

In 2011, the FLAME trial (fluoxetine in motor recovery of patients with acute ischaemic stroke) reported that a 3-month course of fluoxetine given within 5 to 10 days of onset of ischaemic stroke improved motor recovery and increased functional independence compared to placebo [3]. This led to the development of three larger trials of fluoxetine for stroke recovery, which aimed to determine whether fluoxetine given early after stroke (2 to 15 days post-onset, either ischaemic or haemorrhagic stroke) increased functional independence at 6 months [4]. These trials (FOCUS [Fluoxetine or Control Under Supervision], AFFINITY [Assessment oF FluoxetINe In sTroke recoverY] and EFFECTS [Efficacy oF Fluoxetine—a randomisEd Controlled Trial in Stroke]) were collaboratively designed, had the same basic protocol, but with the option of adding additional outcomes. FOCUS published its results among 3127 patients in December 2018: there was no effect of fluoxetine on the primary outcome of modified Rankin scale (mRS), but those allocated to fluoxetine were less likely to develop depression by 6 months and were more likely to have had a bone fracture [5]. AFFINITY (*n* = 1280 [6]) and EFFECTS (*n* = 1500 [7]) published their 6-month data in August 2020. The findings were nearly identical to those in FOCUS: there was no effect of fluoxetine on the mRS, but those allocated to fluoxetine were less likely to develop depression in the EFFECTS trial and were more likely to have had a bone fracture in the AFFINITY and EFFECTS trials.

### Pre-planned individual patient data meta-analysis of FOCUS, AFFINITY and EFFECTS

When the collaboration was initiated, we published a core protocol for the trials and agreed to perform an individual patient data meta-analysis (IPDM) after all three trials had reported their primary results [4].

We considered whether to include other trials of fluoxetine in our IPDM. However, we know from our first Cochrane review and meta-analysis of randomised controlled trials of selective serotonin reuptake inhibitors for stroke recovery [8] that low-quality trials tend to give positive results whilst high-quality trials do not. Although there were two other trials of fluoxetine which were at low risk of bias [3, 9] (recruiting 118 and 32 patients respectively), they were substantially different in their design from FOCUS, AFFINITY and EFFECTS (e.g. different inclusion criteria, shorter duration of treatment and different outcome measures). Thus, we decided to limit our IPDM to FOCUS, AFFINITY and EFFECTS. We have, however, included these other two high-quality trials [3, 9] in a systematic review and traditional meta-analysis of fluoxetine for stroke recovery [10] and in the updated Cochrane systematic review (2019) [11]. When the Cochrane review is updated again, we will include other new trials of fluoxetine for stroke recovery.

There are multiple advantages of IPDM over traditional meta-analysis which combines aggregate effect sizes from each trial [12]. Meta-analysis results for specific subgroups of participants can be obtained across studies and differential (treatment) effects can be assessed across individuals, which can help reduce between-study heterogeneity. An IPDM of our three trials will allow us to provide the most precise estimates of any risks and benefits, to detect reliably a smaller overall effect size than those detectable by the individual trials, to better determine the effects of fluoxetine vs placebo in subgroups and to broaden the generalisability of the results. Also, an IPDM can identify if, and where, the treatment effect of fluoxetine and placebo differs between studies since we hypothesise that the treatment effect might be influenced by characteristics of the local healthcare systems and the local trial processes.

The main drawback of an IPDM is the resources and time needed to obtain the data from the trialists and to perform the analyses. However, our data have been collected in a way to facilitate sharing, and the statistical analyses of the individual trials are performed using the same pre-specified analyses.

We have already published a brief description of the IPDM as part of our statistical analysis plan for the three trials [13]. We now describe in detail the methods for our IPDM, following the standard requirements for the rationale, conduct and reporting of an IPDM [12].

## Aim

We will perform an IPDM of FOCUS, AFFINITY and EFFECTS data. Initially, we will include only the 6-month follow-up data (which includes our primary outcome measure). We will report the 12-month data in a subsequent publication. This decision was made before knowing the results of AFFINITY and EFFECTS. We will therefore be able to expedite the IPDM and thus provide important analyses that will inform clinical practice and help researchers make decisions about whether further trials of SSRI for stroke are needed. The objectives below describe the analyses at 6 months and 12 months.

## Objectives

### Primary objective

We will determine whether patients with a clinical stroke diagnosis (2 to 15 days after onset) who are prescribed a 6-month course of fluoxetine 20 mg daily have improved functional outcome, as defined by the modified Rankin Scale (mRS) score at 6 and 12 months, compared with placebo [4].

Pre-specified secondary objectives (aligning with secondary analyses for the individual trials) [4, 13]:

1. Does fluoxetine influence the secondary outcomes (fatigue, individual stroke impact score domains, cognition, mood, quality of life and living circumstances) at 6 months and 12 months? In addition to the analysis of individual stroke impact score (SIS) domains, we will derive a motor score and a physical function score from relevant individual items [4].
2. If fluoxetine improves mRS score at 6 months, does any improvement persist after treatment stops?
3. Does fluoxetine increase the risk of serious adverse events?
4. Is fluoxetine associated with longer-term survival? The survival analysis will be by the Kaplan-Meier technique and the groups compared by the Cox proportional hazards analysis, expressed as a hazard ratio.
5. Is the effect of fluoxetine vs placebo on the primary outcome modified by any of the following: stroke pathology, age, stroke severity (based on the six variable model and the National Institutes of Health stroke scale (NIHSS)), patients unable to consent for themselves, inability to assess mood because of communication or cognitive problems, and patients with and without depression at baseline?
6. In patients with motor deficits at randomisation, does fluoxetine improve motor function as measured by the SIS motor, and is the effect of fluoxetine modified by the severity of the stroke, as measured by the NIHSS?
7. In patients with aphasia at randomisation, does fluoxetine reduce aphasia?
8. Is there a relationship between functional status at 6 months and mood, and is this relationship affected by fluoxetine?
9. How does non-adherence to the study protocol influence outcome?

Additional pre-specified secondary analyses in the IPDM:

10 Does the effect of fluoxetine vs placebo vary by country of randomisation (UK, Australia, Vietnam, New Zealand, Sweden)? Note that this is not the same as by trial. If we do not have sufficient numbers of patients from one of the countries to ensure that anonymity is preserved, we will perform this analysis by trial only.
11 Does the effect of fluoxetine vary by ethnicity? This will overlap to some extent with country.
12 Does the effect of fluoxetine vary by trial? This will also overlap with country and ethnicity.

#### Data-driven exploratory analyses

Data-driven analyses will be conducted to address heterogeneous treatment effects. The aim is to look at subgroups of patients who may respond differently from others to fluoxetine, e.g. at higher risk of adverse effects, of gaining more benefit etc. This was considered for FOCUS, but there were insufficient data to perform these analyses. Subpopulations with different average treatment effects will be identified using ‘regression tree’ or ‘recursive partitioning’ methods [14]. These data-driven analyses will be identified as such when we report the IPDM results.

#### Resource use and cost-effectiveness: future analyses

Each trial is collecting data about resource use over the first 12 months of follow-up to enable us to carry out health economic analyses [4, 13]; these will be trial-specific, but a combined analysis will be undertaken including data common to all three trials. It will be led by the health economists in our collaborative group and will be reported in a subsequent publication. The statistical analysis plan for the individual trials describes the health economic analyses in more detail [13]. A separate protocol will be developed for these analyses.

## Patient eligibility criteria for inclusion in the IPDM

We will include all the patients who were randomised in the three trials. We will exclude those who consented but were not subsequently randomised.

In FOCUS, there was a small number of patients where the 6-month outcome data were obtained too early (less than 90 days) or too late (more than 1 year from randomisation); in the IPDM, these will be dealt with in the same way as in FOCUS, i.e. if the mRS score was available between 90 days and 1 year, we included it within our primary analysis based on the 6-month follow-up. If it was not between these times, it was not used. If after 1 year, it would have been used for 12-month analyses.

We will do a sensitivity analysis sequentially excluding patients, starting with those who did not meet the entry criteria for the trials. This has already been described in detail in the statistical analysis plan for the individual trials [13]. We will do these sequential exclusions in the same way as we did in FOCUS, which means that those without stroke will be excluded. Note that in FOCUS the numbers were very small.

## Studies to be included in the IPDM

We pre-specified that we would include FOCUS, AFFINITY and EFFECTS in this IPDM. Thus, we are not reporting in this protocol the study selection processes.

## Data collection process

The FOCUS statisticians will merge the data sets from the three trials to produce a combined data set that is ‘future proofed’, to enable new research questions identified in the future to be addressed. The combined data set will include all variables, even if only collected by one or two of the trials.

## Data items

In the IPDM, we will include the core data items (baseline and outcome data) which have been collected in all three trials and any measures that are common to at least two of the individual trials.

The following outcome domains are common to all three trials, but different variables have been collected:

- Cognition. The SIS which incorporates an assessment of memory and thinking that was collected for all three trials. An IPDM will be performed for those data. In AFFINITY, cognition during follow-up is assessed with the Modified Telephone Interview for Cognitive Status (TICSm). EFFECTS assessed cognition with the Montreal Cognitive Assessment (MoCA) at baseline, 3 months and 6 months (face-to-face); these scales will not be merged. When we update the Cochrane review of SSRI for stroke recovery, we will use standardised mean difference (SMD) to combine data from different scales.
- Mood. All three trials report ‘a new diagnosis and/or treatment of depression during follow-up’. FOCUS and EFFECTS also report the Mental Health Inventory 5. These will be combined in the IPDM. There are also data from the PHQ-9 in AFFINITY and MADRS in EFFECTS; these scales will not be combined in this IPDM. When we update the Cochrane review of SSRI for stroke recovery, we will use SMD to combine these scales.

### Data dictionary

We have developed a data dictionary based on FOCUS records, which has ~ 500 variables coded. This will be sent to the AFFINITY and EFFECTS trialists. The FOCUS data dictionary will enable EFFECTS and AFFINITY trialists to align their data dictionaries to FOCUS, so that the same variables are coded in the same way for each trial. Thus, when AFFINITY and EFFECTS data are made available to FOCUS statisticians, minimal manipulation will be required to merge the data sets.

### Transfer of data from AFFINITY and EFFECTS to the FOCUS statistician

After the data dictionaries have been aligned, AFFINITY and EFFECTS trialists will transfer their anonymised data to the FOCUS statisticians. Initially, this will include all data collected up to 6 months. When the 12-month data become available, these will also be provided. The process of data transfer will be determined by the governance processes in each country; this is likely to be using file exchange via secure FTP (SFTP).

### Merging the trial data sets

FOCUS own the FOCUS data. AFFINITY and EFFECTS own their respective data but have given permission for these data to be combined with FOCUS.

The merged data sets will be stored in the University of Edinburgh Data store, along with a detailed data dictionary and descriptors and a description of how the merging was done. FOCUS data will be shared as part of the researchers’ responsibilities to the funder. Further discussion is needed about whether, and if so, how, to share the merged data set with other researchers; we will follow the recommendations of the three funders and the institutions which hosted the three trials.

Whilst the statistician performing the IPDM works on the combined data set, it will be password protected and the analysis will be done in a locked office. SAS will be used for the statistical analysis. Note that the health economic IPDM may be performed using different programmes.

## Tables of results for IPDM

We will report baseline data for the three trials individually and together, the outcome measures for the individual trials and the combined data set, and the subgroups of interest for the individual trials and the combined data set (Table 1 in the Appendix, Table 2 in the Appendix and Table 3 in the Appendix). These align with our research questions.

## Risk of bias assessment in individual studies

The risk of bias for each trial will be assessed by two independent people outside of the author team using the Cochrane risk of bias tool 2 [15]. These two people will be identified after the publication of this protocol. They will be independent of the trialists and will have had previous experience of using either the first or second Cochrane risk of bias tool.

## Specification of outcomes and effect measures

The outcomes listed in the statistical analysis plan for the three trials [13] are the mRS score, the SIS, the vitality component of SF-36 (for fatigue), mood, EQ5D-5L (quality of life), adverse events and living circumstances.

## Synthesis methods

### Descriptive analyses of the three trials (Table 1 in the Appendix)

Descriptive and exploratory analyses will be undertaken initially to identify and display differences in baseline characteristics between the types of patients enrolled in the three trials, specifically statistical comparisons of baseline means and medians. We will use ANOVA to explore whether there is evidence of a difference across trials. Also, we will describe the duration and type of hospital stays between randomisation and discharge home, discharge to a residential or nursing home, or death.

Each trial will be re-analysed individually to ensure that the main results can be reproduced. If that is not the case, the differences must be resolved before proceeding with the IPDM meta-analysis.

### IPDM

We have considered whether to do a one- or two-stage IPDM [16]. There are advantages and disadvantages to both approaches. A one-stage model combines all patient data from all studies in one single model and takes advantage of the combined data set. It also gives greater flexibility and power than the alternative two-stage method but can be more complex and difficult to interpret. The two-stage method involves the creation of summary statistics then combining the summary statistics using standard meta-analysis methods, but the drawback of this approach is that it can be biased.

We have decided to do both approaches as a sensitivity analysis. The one-stage approach will provide estimates of effect, and the two-stage approach will provide Forest plots. Generally, the two approaches should give the same answer [16], but if the approaches give different answers, we will explore the reasons for this. In general, different answers from the two methods tend to be obtained when included studies are small and/or when an effect is large, or events are rare [16]. This is unlikely to be the case for this IPDM as the three studies are large. Furthermore, the effects in FOCUS were small, and results from EFFECTS and AFFINITY are awaited.

#### One-stage approach

We will perform the same analyses that have been described for the individual trials on the combined data set. This has been described in the FOCUS trial publication [5]. This will be a regression model, with a separate term for each trial.

#### Two-stage approach

Ordinal logistic, logistic and Cox proportional hazards regressions will be performed separately for each trial, as described for the main within-trial analysis. Then, the point estimate and standard error of the treatment effect from the within-trial analyses will be meta-analysed using a generic inverse variance model. Fixed effect analyses will be performed and presented as there are only three studies and so the estimate of the between-study variability will be poor. Forest plots will be produced to display results.

## How missing data within the IPDM will be dealt with

Our randomisation systems did not allow investigators to proceed to treatment allocation without entering complete baseline data. The mRS, our primary outcome, includes death, so it is expected that the number with missing mRS data at follow-up will be minimal. The primary analysis will be a complete case analysis (i.e. anyone with missing mRS data will not be included in any analysis requiring mRS). Multiple imputations will only be performed if the proportion of data missing is > 5%.

For secondary outcomes where missing data are expected because data will not be available for patients who did not survive, we will present results for those who are alive at follow-up; any discrepancy in death rates between groups will be considered in the interpretation. Missing data for single questions within scores will be handled as described by each scoring method. Immediately before database lock for each trial, a blinded review of the completeness of primary and secondary outcomes is performed. For items such as SF36, if there are at least 50% of questions within a domain that have responses, we are able to use the methods detailed by the standardised tools to calculate a value for the domain.

If higher levels of missing mRS data than expected (> 5%) are seen, we will consider additional sensitivity analyses to assess the robustness of the results against various assumptions about the missing data mechanism as outlined in Jakobsen et al. [17]. Given the exact nature of these additional sensitivity analyses is not pre-specified, they will be considered exploratory.

### Primary analysis

This addresses the primary research question: does the routine administration of fluoxetine 20 mg daily for 6 months after an acute stroke improve patients’ functional recovery at 6 months?

### Secondary analyses

#### Secondary outcomes

We will report the secondary outcomes at 6 and 12 months for the combined data set, as we have done for the individual trials and as described in the statistical analysis plan for the individual trials.

#### Subgroup analyses

We will perform subgroup analyses as we have done for the individual trials. The mRS data at 6 months will be compared using an ordinal logistic regression using our adjusted model, and comparison will be made across variables listed in Table 1 in the Appendix. Each subgroup will be considered separately by adding the subgroup variable and its interaction with the treatment variable to the main model. This includes examining individual and group covariates of substantive interest such as stroke severity (NIHSS) and the six simple variable model for prognosis [4]. Potential covariates and other baseline data are shown in Table 1 in the Appendix. Additionally, we will explore the impact of trial (FOCUS, AFFINITY, EFFECTS), country and ethnicity. This will enable us to explore variation in effects.

#### Sensitivity analyses

We will sequentially exclude patients from the trials to explore the influence of non-eligible patients being recruited, and compliance with the trial medication as already described in detail in the statistical analysis plan for the individual trials [13].

### Data-driven (additional) analyses

Data-driven analyses will be conducted to address heterogeneous treatment effects. Subpopulations with different average treatment effects will be identified using ‘regression tree’ or ‘recursive partitioning’ methods [14]. These data-driven analyses will complement the pre-specified subgroup analyses.

## Risk of bias across studies

We will rate the certainty of the evidence of the IPDM using Cochrane Grades of Recommendation, Assessment, development, and Evaluation (GRADE) [18].

## Reporting of results

We will produce a PRISMA IPDM diagram; this will not include results of searches for studies as we have pre-specified that we are using only data from three trials. We will list available data and analysed data (i.e. the final two steps in a standard PRISMA IPDM diagram) [19].

We will report results in individual trials and results in the combined data set (the one-stage approach) as shown in results Table 1 in the Appendix, Table 2 in the Appendix and Table 3 in the Appendix for baseline, subgroup and pre-specified outcomes.

We will produce Forest plots for the two-stage approach (i.e. where trials are analysed independently and then meta-analysed) for all the pre-specified outcomes. However, there are likely to be too many plots to present in a publication. As a minimum, we will include the plot for the primary outcome, which will include effects in the individual pre-specified subgroups. We will describe the other outcomes in the text as well as making them available on reasonable request, or in an appendix, depending on the journal requirements.

The text will report study characteristics, IPDM integrity, risk of bias within studies, results of individual studies (including the impact of compliance on the results), results of syntheses, risk of bias across studies and any additional analyses performed.

## Discussion of IPDM

In the discussion, we will summarise evidence, its strengths and limitations, how it fits with existing literature, our conclusions and the implications for clinical practice, future research and policy.

## Further possible analyses of the combined data set

There are several other research questions that can be addressed using the combined data set. Each of these questions will require a separate statistical analysis plan to be developed. These questions include:

- What are the types of bone fractures after stroke? What is their impact on mRS? What factors are associated with fracture risk? We have already published these data from FOCUS and can repeat the same analyses on the combined data set. Note that falls will be included in the interpretation of these results.
- What are the baseline predictors of seizure risk after stroke? What drugs are associated with increased seizure risk? How does fluoxetine modify this effect?
- What are the baseline predictors of depression after stroke? How is depression at 6 months associated with disability and dependency?
- What factors are associated with pain after stroke (using pain item from EQ5D-5L)? This includes baseline predictors and factors that might influence pain severity, e.g. mood disorders.
- What are the baseline predictors of fatigue after stroke? Fatigue is measured using the SF-36 vitality component. How does fatigue relate to mood and does it predict case fatality?
- How does the short version of the Stroke Impact Scale (using a single item from each domain) relate to the full Stroke Impact Scale? This is an important question for researchers who are considering using the Short Stroke Impact Scale in future research.
- What is the effect of fluoxetine on 90-day home-time, and what is the relationship between home-time and mRS across countries? Home-time is a measure of the time spent back at the person’s home during a predefined period after a stroke [20].

## Trial status

FOCUS recruited its first patient on 10 September 2012 and its last on 31 March 2017 and published its results in December 2019.

AFFINITY recruited its first patient on 11 January 2013 and its last patient on 30 June 2019.

EFFECTS recruited its first patient on 20 October 2014 and its last patient on 28 June 2019.

## Data Availability

The data sets generated and/or analysed during the current study are not currently publicly available because we wish to perform and publish our own analyses initially but may be available in due course from the corresponding author on reasonable request. The EFFECTS data sets used can be made available by EL on reasonable request. However, according to the Swedish Secrecy Act 24:8, an interested researcher first must apply and receive approval from the Swedish Ethical Review Authority. Written proposals will be assessed by the EFFECTS steering committee and a decision made about the appropriateness of the use of data. A data sharing agreement will be put in place before any data are shared.

## Appendix

As stated in the initial statistical analysis plan, we will show data from all three trials as well as data from the merged data set. However, the table will be large and so data from the individual trials may need to be included in an appendix, and only data from the merged data set included in the main part of the paper.

The following table is based on the assumption that all the data are present; where this is not the case, denominators will be indicated to provide the reader with a clear idea of the numbers that each percentage was calculated from.

Note: this table is different from the data dictionary. The data dictionary is a separate document that is about 40 pages long and lists ~ 500 variables.

**Table 1:** Baseline data collected before randomisation in the three trials

		FOCUS (N= 3127)			AFFINITY (N=1280)			EFFECTS (N=1500)			Combined (N=5907)		
		Fluoxetine (n=1564)	Placebo (n= )	All (n= )	Fluoxetine (n= )	Placebo (n= )	All (n= )	Fluoxetine (n= )	Placebo (n= )	All (n= )	Fluoxetine (n= )	Placebo (n= )	All (n= )
Sex	Women	N(%)											
	Men	N(%)											
Age	Age ≤70 years	N(%)											
	Age >70 years	N(%)											
	Mean (sd)	Mean (sd)											
Ethnicity	Asian	N(%)											
	Black	N(%)											
	Chinese	N(%)											
	White	N(%)											
	Other	N(%)											
Marital Status	Married	N(%)											
	Partner	N(%)											
	Divorcer/separated	N(%)											
	Widowed	N(%)											
	Single	N(%)											
	Other	N(%)											
Living arrangement	Living with someone	N(%)											
	Living alone	N(%)											
	Living in an inst.	N(%)											
	Other	N(%)											
Employment	FT	N(%)											
	PT	N(%)											
	Retired	N(%)											
	Unemployed/disabled	N(%)											
	Other	N(%)											
Independent before stroke		N(%)											
Previous medical history	Coronary heart disease	N(%)											
	Ischaemic stroke/TIA	N(%)											
	Diabetes	N(%)											
	Hyponatraemia	N(%)											
	Intracranial bleed	N(%)											
	Upper GI bleed	N(%)											
	Bone fractures	N(%)											
	Depression	N(%)											
Stroke diagnosis	Not-stroke	N(%)											
	Ischaemic stroke	N(%)											
	Intracerebral haemorrhage	N(%)											
OCSP classification of Ischaemic stroke	Total anterior circulation infarct	N(%)											
	Partial anterior circulation infarct	N(%)											
	Lacunar infarct	N(%)											
	Posterior circulation infarct	N(%)											
	Uncertain	N(%)											
Cause of stroke, modified TOAST classification	Large artery disease	N(%)											
	Small vessel disease	N(%)											
	Embolism from heart	N(%)											
	Another cause	N(%)											
	Unknown/uncertain	N(%)											
Predictive variables	Able to walk at time of randomisation	N(%)											
	Able to lift both arms off bed	N(%)											
	Able to talk and not confused	N(%)											
Predicted 6-month outcome based on SSV	Prob. of being alive and independent	Q2(Q1,q3)											
	0.00 to ≤ 0.15	N(%)											
	>0.15 to 1.00	N(%)											
Neurological deficits	NIHSS	Q2(Q1,q3)											
	Presence of motor deficit	N(%)											
	Presence of aphasia	N(%)											
Depression at baseline	Current diagnosis of depression	N(%)											
	Taking a non-SSRI antidepressant	N(%)											
Current mood (PHQ-2)	2 yes responses	N(%)											
	1 yes response	N(%)											
	0 yes responses	N(%)											
Delay (days) since stroke onset at randomisation	Mean (sd)	Mean (sd)											
	2-8 days	N(%)											
	9-15 days	N(%)											
Details of enrolment	Enrolled as a hospital inpatient	N(%)											
	Patient consented	N(%)											
	Proxy consented	N(%)											

*Abbreviations*: *FT* full time, *GI* gastrointestinal, *NIHSS* National Institutes of Health stroke scale, *OCSP* Oxfordshire Community Stroke Project, *PHQ-2* Patient Health Questionnaire 2-item, *PT* part time, *sd* standard deviation, *SSRI* selective serotonin reuptake inhibitor, *SSV* six simple variables, *TIA* transient ischaemic attack, *TOAST* Trial of ORG 10172 in Acute Stroke Treatment

**Table 2:** Outcome variables including adverse events and coding at 6 months (same table for 12 months without the AE section once the 12-month data becomes available from all trials but this will be a separate paper)

		FOCUS (N= )			AFFINITY (N= )			EFFECTS (N= )			Combined (N= )		
		Fluoxetine (n=)	Placebo (n=)	All (n=)	Fluoxetine (n= )	Placebo (n= )	All (n= )	Fluoxetine (n= )	Placebo (n= )	All (n= )	Fluoxetine (n= )	Placebo (n= )	All (n= )
Modified Rankin Score	0 - No Symptoms	N(%)											
	1	N(%)											
	2	N(%)											
	3	N(%)											
	4	N(%)											
	5	N(%)											
	6 – Dead	N(%)											
Stroke Impact Scale (SIS)	Strength	Q2(Q1,q3)											
	Hand ability	Q2(Q1,q3)											
	Mobility	Q2(Q1,q3)											
	Motor	Q2(Q1,q3)											
	Daily activities	Q2(Q1,q3)											
	Physical function	Q2(Q1,q3)											
	Memory	Q2(Q1,q3)											
	Communication	Q2(Q1,q3)											
	Emotion	Q2(Q1,q3)											
	Participation	Q2(Q1,q3)											
	Recover (VAS)	Q2(Q1,q3)											
	Motor score derived from three domains (arm, hand, leg and foot strength; hand function and mobility)	Q2(Q1,q3)											
	Physical function score derived from four domains (arm, hand, leg and foot strength; hand function; mobility; Daily activities)	Q2(Q1,q3)											
Vitality		Q2(Q1,q3)											
Mood	MHI in FOCUS and EFFECTS PHQ-9 in AFFINITY MADRS in EFFECTS	Q2(Q1,q3)											
EQ5D-5L		Q2(Q1,q3)											
Adverse events	Any stroke	N(%)											
	All thrombotic events	N(%)											
	Ischaemic stroke	N(%)											
	Other thrombotic events	N(%)											
	Acute coronary events	N(%)											
	All bleeding events	N(%)											
	Haemorrhagic stroke	N(%)											
	Upper GI bleed	N(%)											
	Other major bleeds	N(%)											
	Epileptic seizures	N(%)											
	Fall with injury	N(%)											
	Fractured bone	N(%)											
	Hyponatraemia	N(%)											
	Hyperglycaemia	N(%)											
	Symptomatic hypoglycaemia	N(%)											
	New depression	N(%)											
	New antidepressant	N(%)											
	Attempted or actual suicide	N(%)											
Living circumstances	Own home	N(%)											
	Relatives	N(%)											
	Care/nursing home	N(%)											
	Long term NHS	N(%)											
	In hospital	N(%)											
	Other	N(%)											

*Abbreviations*: *EQ5D-5L* EuroQol 5 dimension questionnaire—five level, *GI* gastrointestinal, *MADRS* Montgomery-Åsberg Depression Rating Scale, *MHI* Mental Health Inventory, *NHS* National Health Service, *PHQ-9* Patient Health Questionnaire 9-item, *VAS* visual analogue scale

Plot similar to this with all trials and an overall effect (one-stage IPDM)

**Table 3:** Subgroup analyses of the individual trials and combined data set (one-stage IPDMM)

		FOCUS			AFFINITY			EFFECTS			Combined		
		Fluoxetine (n=)	Placebo (n=)	COR (95% CI) p-value	Fluoxetine (n=)	Placebo (n=)	COR (95% CI) p-value	Fluoxetine (n=)	Placebo (n=)	COR (95% CI) p-value	Fluoxetine (n=)	Placebo (n=)	COR (95% CI) p-value
Probability of being alive and independent at 6 months using SSV	0 to ≤0∙15												
	0.15-1.00												
Delay from onset to randomisation	2-8 days												
	9-15 days												
Motor deficit	No												
	Yes												
Aphasia	No												
	Yes												
Stroke type	Haemorrhagic												
	Ischaemic												
Age group	≤70 years old												
	>70 years old												
Who gave consent	Proxy												
	Patient												
Inability to assess mood	No												
	Yes												
Baseline depression	No												
	Yes												
Country	UK												
	Vietnam												
	New Zealand												
	Australia												
	Sweden												
Ethnicity	Asian												
	Black												
	Chinese												
	White												
	Other												
Trial	FOCUS												
	AFFINITY												
	EFFECTS												

*Abbreviations*: *CI* confidence interval, *COR* common odds ratio, *SSV* six simple variables

## Abbreviations

AFFINITY: Assessment oF FluoxetINe In sTroke recoverY
CI: Confidence interval
COR: Common odds ratio
EFFECTS: Efficacy oF Fluoxetine—a randomisEd Controlled Trial in Stroke
EQ5D-5L: EuroQol 5 dimension questionnaire—five level
FOCUS: Fluoxetine Or Control Under Supervision
GI: Gastrointestinal
IPDM: Individual patient data meta-analysis
MADRS: Montgomery-Åsberg Depression Rating Scale
MHI5: Mental Health Inventory 5 questions
MoCA: Montreal Cognitive Assessment
mRS: Modified Rankin scale questionnaire
NIHSS: National Institutes of Health stroke scale
OCSP: Oxfordshire Community Stroke Project
PHQ: Patient Health Questionnaire
RR: Relative risk
SF36: 36-Item Short Form Health Survey
SIS: Stroke Impact Scale
SMD: Standardised mean difference
SSRI: Selective serotonin reuptake inhibitor
SSV: Six simple variables
TICSm: Modified Telephone Interview for Cognitive Status
TOAST: Trial of ORG 10172 in Acute Stroke Treatment
UK: United Kingdom
